# Serotonin, Dopamine and Noradrenaline Adjust Actions of Myelinated Afferents via Modulation of Presynaptic Inhibition in the Mouse Spinal Cord

**DOI:** 10.1371/journal.pone.0089999

**Published:** 2014-02-28

**Authors:** David L. García-Ramírez, Jorge R. Calvo, Shawn Hochman, Jorge N. Quevedo

**Affiliations:** 1 Departamento de Fisiología, Biofísica y Neurociencias, Centro de Investigación y de Estudios Avanzados del Instituto Politécnico Nacional, México, D.F., México; 2 Department of Physiology, Emory University, Atlanta, Georgia, United States of America; The Research Center of Neurobiology-Neurophysiology of Marseille, France

## Abstract

Gain control of primary afferent neurotransmission at their intraspinal terminals occurs by several mechanisms including primary afferent depolarization (PAD). PAD produces presynaptic inhibition via a reduction in transmitter release. While it is known that descending monoaminergic pathways complexly regulate sensory processing, the extent these actions include modulation of afferent-evoked PAD remains uncertain. We investigated the effects of serotonin (5HT), dopamine (DA) and noradrenaline (NA) on afferent transmission and PAD. Responses were evoked by stimulation of myelinated hindlimb cutaneous and muscle afferents in the isolated neonatal mouse spinal cord. Monosynaptic responses were examined in the deep dorsal horn either as population excitatory synaptic responses (recorded as extracellular field potentials; EFPs) or intracellular excitatory postsynaptic currents (EPSCs). The magnitude of PAD generated intraspinally was estimated from electrotonically back-propagating dorsal root potentials (DRPs) recorded on lumbar dorsal roots. 5HT depressed the DRP by 76%. Monosynaptic actions were similarly depressed by 5HT (EFPs 54%; EPSCs 75%) but with a slower time course. This suggests that depression of monosynaptic EFPs and DRPs occurs by independent mechanisms. DA and NA had similar depressant actions on DRPs but weaker effects on EFPs. IC_50_ values for DRP depression were 0.6, 0.8 and 1.0 µM for 5HT, DA and NA, respectively. Depression of DRPs by monoamines was nearly-identical in both muscle and cutaneous afferent-evoked responses, supporting a global modulation of the multimodal afferents stimulated. 5HT, DA and NA produced no change in the compound antidromic potentials evoked by intraspinal microstimulation indicating that depression of the DRP is unrelated to direct changes in the excitability of intraspinal afferent fibers, but due to metabotropic receptor activation. In summary, both myelinated afferent-evoked DRPs and monosynaptic transmission in the dorsal horn are broadly reduced by descending monoamine transmitters. These actions likely integrate with modulatory actions elsewhere to reconfigure spinal circuits during motor behaviors.

## Introduction

Descending monoaminergic transmitter systems (5HT, NA and DA) play an integral role in modulating spinal sensory processing, capable of both depression and facilitation of sensory evoked actions (see [1], [2], [3], [4], [5]). A powerful mechanism used to depress afferent input strength is via PAD produced predominantly following activation of GABA_A_ receptors on primary afferent terminals [6]. PAD production is widespread and generated by a highly differentiated spinal circuitry, highlighting its important contribution to the regulation of sensory neurotransmission. An important question is the extent to which monoaminergic pathways regulate afferent activity by controlling PAD. While the monoamines are involved in PAD of predominantly pain/temperature encoding afferents (e.g. [7], [8], [9]), little is known about their modulation on PAD of proprioception-encoding muscle afferents [10], [11], [12], [13] with no reported effects on myelinated cutaneous pathways [14], [15], [16].

Stimulation of brainstem raphespinal pathways regulates PAD of group Ia muscle spindle and Ib Golgi tendon organ afferents [13], [17] but whether these actions are due to direct effects of 5HT is not known. Facilitation of group I muscle afferent actions, presumably on interneurons contacted monosynaptically by these afferents, were observed by local injection of 5HT and NA near different spinal neurons [1], [18], [19]. Conversely, actions on afferent fibers are implicated in the monoaminergic depression of Ia muscle afferent-evoked monosynaptic reflexes [20], [21], [22], [23].

Stimulation of coerulospinal and raphespinal brainstem nuclei produces PAD on low-threshold group II muscle spindle afferent terminals [12]. Actions via the spinal release of monoamines are suggested, as their local application can depress group II muscle afferent-evoked population EPSPs seen as extracellular field potentials (EFPs) [24], [25], [26]. However, EFP depression can be independent of PAD, instead produced by direct metabotropic receptor actions on afferent terminals [27], so the relation between PAD and release of monoamines from descending terminals remains uncertain.

Overall, while suggestive, there is currently no direct evidence of the monoamines modulating myelinated ‘low-threshold’ sensory-evoked PAD. Accordingly, we applied 5HT, DA, and NA to study their actions on sensory evoked-DRPs in the isolated mouse spinal cord with intact hindlimb muscle and cutaneous nerves. This approach allows study of intact afferent pathways *in vitro* in neonates while providing enhanced recording stability and more precise pharmacological control [28]. In addition, we evaluated whether modulatory actions occur directly on the first-order synapse initiating activation of PAD pathways by recording monosynaptic EFPs. We also measured changes in excitability of the afferent fibers that evoke PAD with intraspinal microstimulation to disclose direct effects by possible ionotropic mechanisms. We found that 5HT, DA and NA most profoundly depressed DRPs via actions on spinal interneurons. Additionally 5HT and NA also depressed monosynaptic afferent transmission. Preliminary data were published in abstract form [29], [30].

## Methods

### Ethics Statement

All the procedures described here comply with the guidelines contained in the National Institutes of Health *Guide for the Care and Use of Laboratory Animals* (USA) and were approved by the Institutional Animal and Use Committee in the Center for Research and Advanced Studies (Mexico).

### Dissection

Experiments were carried out in 6-7-day-old BALB/c mice of both sexes. Animals were anesthetized with 10% urethane (2 mg/kg i.p.) before decapitation at cervical level. The thoracic to lumbar cord was exposed by dorsal and ventral approach in cooled high-sucrose solution containing (in mM): sucrose 250; NaCl 125; KCl 2.5; D-glucose 25; MgS0_4_ 3.0; CaCl_2_.2H_2_O, 1.0; NaH_2_PO_4_.H_2_O 1.25; and NaHCO_3_ 26. The dorsal laminectomy and the ventral vertebrectomy were performed with special care to maintain paravertebral muscles and both dorsal and ventral spinal roots in continuity with peripheral nerves. In some experiments pelvis and hindlimbs were maintained *in situ* and nerves dissected free. In other experiments the sciatic nerve and branches were dissected free and the rest of the limb was removed. The preparation was pinned ventral side down in a Sylgard-coated Petri dish. A sagittal hemisection of the cord was performed by means of insect pins and each hemisected cord was transferred to a separate dish. Peripheral nerves tibial (Tib), deep peroneal (DP), semitendinosus (St), posterior biceps (PB), superficial peroneal (SP) and caudal cutaneous sural (Su) were dissected free and sectioned distally. A hemisected cord with hindlimb attached, or with dissected sciatic nerve and branches, was transferred to a 5 ml bath chamber with artificial cerebrospinal fluid (ACSF) containing (in mM): NaCl 125; KCl 2.5; D-glucose 25; MgS0_4_ 1.0; CaCl_2_.2H_2_O, 2.4; NaH_2_PO_4_.H_2_O 1.25; and NaHCO_3_ 26. The ACSF was perfused via recirculation at 8–10 ml/min, and was maintained at 23°C and gassed with 95% O_2_/5% CO_2_. The hemisected cord was stabilized by means of a hammock built with crossed insect pins underneath the cord, and the cut surface of the cord was positioned with an angle of 45°. The preparation was then allowed to recover for about 1 h before any other manipulation.

### Stimulation and recording

**Figure 1:**
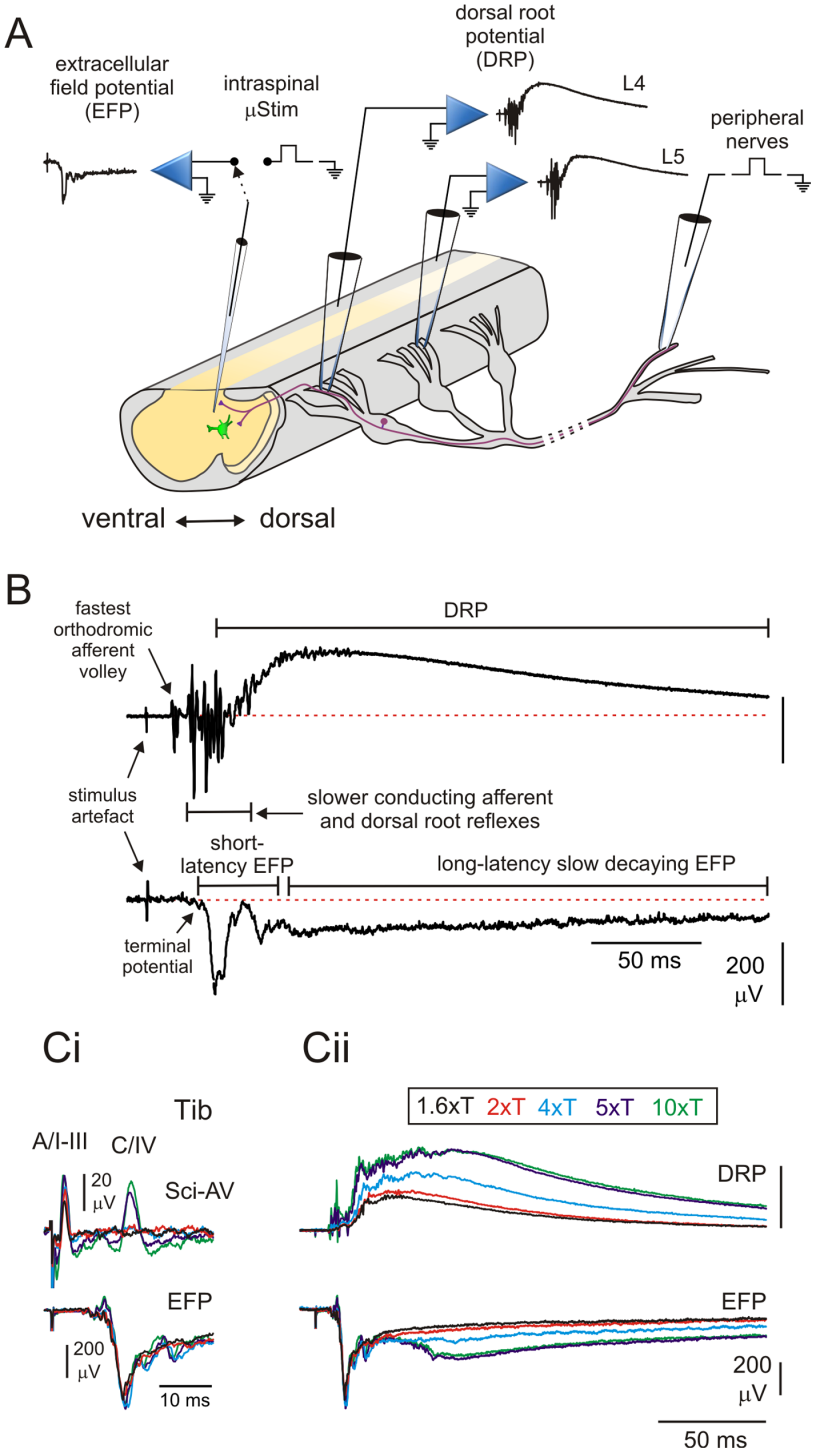
Methods. **A**, schematic representation of the experimental setup showing the hemisected spinal cord and the sciatic nerve with dissected nerve branches. Extracellular field potentials (EFPs) were recorded in the deep dorsal horn at L4. Microstimulation (μStim) was applied through the same micropipette where the evoked-EFPs were larger. Dorsal root potentials (DRPs) were recorded in dorsal roots L4 and L5. Peripheral nerves were stimulated at strengths based on the recruitment of the most excitable fibers. **B**, DRP (*upper trace*) and EFP (*lower trace*) produced by stimulation of the tibial nerve with strength 4 xT. By convention DRPs are presented with negativity upwards and EFPs with negativity downwards. **C**. Comparison of evoked responses observed at different stimulation strengths. **Ci**, afferent volley (*upper traces*) recorded in the sciatic nerve (Sci-AV) and EFP (*lower traces*) recorded at L4 by stimulation of the tibial (Tib) nerve. The fastest component of the sciatic afferent volley corresponds to myelinated (A cutaneous/I-III muscle) and the second one to unmyelinated afferents (C cutaneous/IV muscle). Note that at 4xT there is no evidence of a C fiber volley. **Cii**, DRP (*upper traces*) and EFP (*lower traces*) recorded at L4 level. Tib was stimulated with graded strengths from 1.6-10 xT. The short-latency EFP is maximal at 2 xT.

The experimental setup is shown schematically in Figure 1A with example traces that identify components of recorded potentials in Figure 1B. Suction electrodes were used to stimulate peripheral nerves, usually one at a time. In some experiments we also used bipolar silver hook electrodes (insulated, except in the tip). For isolation from surrounding ACSF, mounted nerves were embedded in petroleum jelly (Vaseline). To limit current spread to adjacent nerves, we always stimulated each nerve as caudal as possible.

Peripheral nerves were stimulated electrically at 0.1 Hz with (0.2 ms pulse duration) at between 1.5–4 times the threshold intensity that recruited the most excitable afferent fibers (xT) in the dorsal root. When afferent fiber compound action potential volleys were recorded in the sciatic nerve, two separate components were observable as described previously in neonatal rodents [31], [32]. The first corresponds to incompletely myelinated afferents (Aβ and Aδ cutaneous/group I–III muscle) and the second to unmyelinated afferents (C cutaneous/group IV muscle) (Figure 1Ci). Unlike the adult where electrical stimulation intensities can discriminate between large, medium and small-diameter myelinated afferents, in the present P6-7 population we could not differentiate between Aβ/I-II and Aδ/III afferent fiber populations based on threshold or conduction velocity [31]. In the example shown in Figure 1Ci, at 4 xT there is no evidence of a C fiber volley. By 5 xT the myelinated axon volley is maximal, with the long-latency C fiber volley being maximal at 10 xT. The long-latency afferent volley clearly represents unmyelinated C fibers based on; (i) measured conduction velocity in the sciatic nerve (0.31 m/s in Figure 1Ci), (ii) the lack of additional volleys at supramaximal current intensities (1 mA, 1 ms), and (iii) its reversible loss following application of capsaicin 100 nM (not shown).

EFPs were recorded in dorsal horn with micropipettes (1–2 MΩ) filled with 2 M NaCl. Micropipettes were placed perpendicularly through the cut surface of the L4-5 spinal segment of the cord, between the central canal and the border of the dorsal column (Figure 1A) at depths between 100–180 µm taking into account the largest amplitude EFPs (approximately between III–VI Rexed laminae). The short-latency EFPs reflect the population monosynaptic excitatory postsynaptic potentials (Figure 1B and C) and were preceded by terminal potentials, that is, the action potentials arriving to all terminals of the stimulated nerve. Figure 1C also shows that the short-latency EFPs are maximal at 2 xT and that the longer slow-decaying EFPs and DRPs are maximal at 5 xT. The longer slow-decaying EFPs reflect the field potential of PAD recorded intraspinally. Based on these observations, we chose 4 xT in most of the experiments to examine near-maximal myelinated fiber-evoked DRP and EFP responses.

‘Blind’ whole-cell voltage-clamp recordings were made to study synaptic actions in individual dorsal horn neurons activated monosynaptically by myelinated afferents. Recording micropipettes contained (in mM):120 CsF, 10 EGTA, 10 HEPES, 10 CsCl_2_, 35 CsOH, and 5 QX314 (to block spiking) at pH 7.3. Micropipette resistance ranged from 2–5 MΩ. Synaptic responses were recorded at several membrane potentials to verify evoked responses as EPSCs. EPSC amplitudes were then measured at potentials where IPSCs reversed (∼−40 mV) before, during and after drug application. We targeted interneurons in locations exhibiting the largest EFPs evoked by nerve stimulation at <2 xT. Mephenesin (1 mM) was applied to isolate the monosynaptic component of EPSCs. Mephenesin has been used to isolate monosynaptic from polysynaptic components in the central nervous system by decreasing the firing threshold of interneurons, leaving the monosynaptic components unaffected [33], [34].

**Figure 2:**
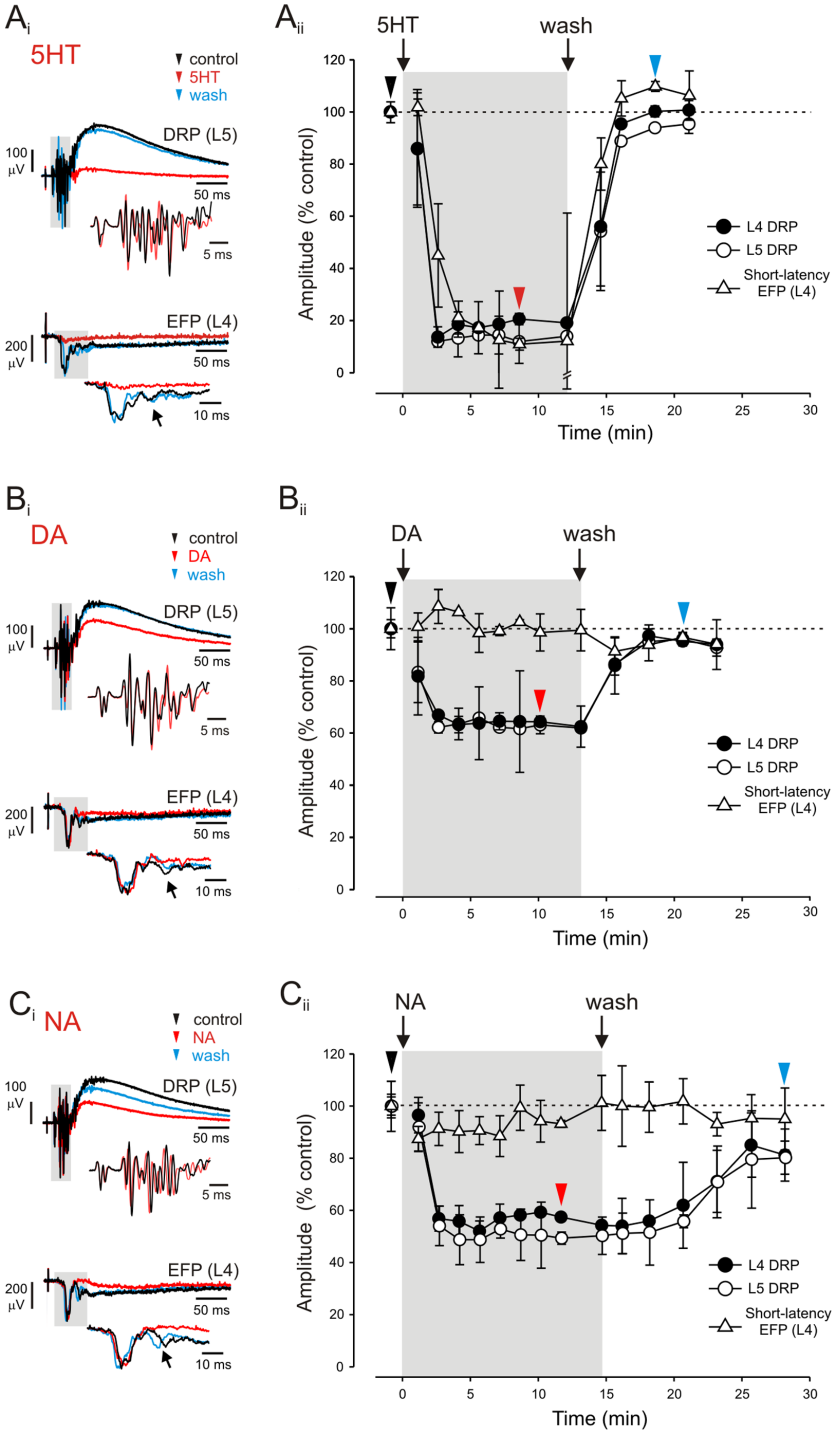
Modulatory actions of monoamines on EFPs and DRPs evoked by stimulation of low-threshold afferents. **Ai**, DRPs (*upper panel*) recorded at L5 dorsal root and EFPs (*lower panel*) recorded in the dorsal horn at L4 before (black), during (red) and after (blue) bath application of 10 µM 5HT (traces are averages of 16 samples). Expanded segments of DRPs and EFPs are shown in insets. EFPs were recorded in the dorsal horn at 120 µm depth from the cut surface of the hemisected cord. The Tib nerve was stimulated at strength 4 xT. Note the remarkable depression of DRPs and short-latency EFPs in the presence of 5HT and the complete recovery after wash. Aside from the clear reversible depression of the larger short-latency EFP, the second short-latency (arrow) and the long-latency slow decaying EFP are also depressed. **Aii**–**Cii**, population averages of time course of monoamine-induced depression of evoked responses. Gray shadows indicate the period of bath application of monoamines. **Aii**, plot of the time course of effects produced by 5HT on L4-DRP (close circles), L5-DRP (open circles) and short-latency EFPs (open triangles). Arrows indicate the time of bath application of 5HT and the onset of drug wash out. Arrowheads point out the time corresponding to traces illustrated in Ai. Each point represents mean ± SD of 16 consecutive events for EFP and DRP amplitude with respect to control. **Bi**–**Bii** and **Ci**–**Cii**, description is identical to Figure Ai and Aii except that DA and NA actions are presented, respectively. Note the clear depression of DRPs in the presence of DA and NA and the incomplete recovery after NA wash. Short-latency EFPs are not significantly affected neither by DA nor NA but the smaller second short-latency (arrows) and the long-latency slow decaying EFP are depressed by these monoamines.

PAD was inferred from dorsal root potentials (DRPs) recorded at L4 and L5 dorsal roots by means of bipolar glass suction electrodes (∼120 µm of tip internal diameter) placed *en passant* on dorsal roots near the entry zone. The short-latency components recorded from these roots correspond to the orthodromically-arriving afferent volleys. These afferent volleys were not affected by the application of monoamines (Figure 2; see expanded traces in insets of L5 DRPs).

Changes in intraspinal afferent fiber excitability was determined using microstimulation (400 µs, ∼10 µA) through the same electrodes used for recording EFPs. This approach, termed Wall's technique, measures changes in afferent terminal excitability [35]. Intraspinal stimulation directly recruits afferent axons whose antidromically-propagated compound action potentials (CAPs) are recorded in dorsal roots or peripheral nerves. We measured the amplitude of the biphasic fastest component of CAPs (peak to peak), which exhibited a mean latency of 1.6 ms (±0.37 S.D.) and were insensitive to block of glutamatergic transmission. During PAD, the same stimulus recruits more fibers to generate a larger CAP because their depolarized terminals are closer to threshold; hence a larger antidromic response indicates an increase in PAD. A lack of change of CAP amplitude, for instance in the presence of monoamines means that the excitability of fast-conducting afferent fibers was not affected. Intraspinal activation of afferent terminals also produces a DRP via orthodromic activation of the circuitry mediating PAD. Both responses were recorded in the L4 and/or L5 dorsal roots.

EFPs and EPSCs were recorded with an Axoclamp 2B amplifier (Molecular Devices, U.S.A.) filtered at 2 kHz, and DRPs with custom made AC-coupled amplifiers (band pass 0.1 Hz–3 KHz), or with DC-coupled amplifiers (A-M Systems, U.S.A; band pass DC – 3 KHz). Raw data were collected with pClamp software (v. 10.0, Molecular Devices, U.S.A.) and stored for off-line analysis.

### Drug solutions and applications

Stock solutions of drugs (10–100 mM) were made and stored at −20°C until needed. All drugs were dissolved in normal ACSF and superfused from separate gravity-fed reservoirs at known concentrations for fixed periods of time over the hemisected cord preparation. Serotonin (5HT), dopamine (DA), noradrenaline (NA) (all from Sigma-Aldrich) were superfused for 10–30 minutes each, depending on the protocol, at a concentration of 10 µM. Mephenesin (Sigma) was superfused for 10 minutes at a concentration of 1 mM. Cumulative dose-response curves for monoamines were made at concentrations of 0.001, 0.01. 0.1, 1, 10 and 100 µM.

### Sample populations

The effects of 5HT, DA and NA were tested in 28 experiments (in 9 experiments only DRPs were recorded; in 19 experiments both DRPs and EFPs) (Table 1). In 20 experiments DRPs were obtained by stimulation of the Tib nerve, while the other 8 experiments involved stimulation of various additional nerves: SP (7), Su (6), DP (4), PB (4) and St (2). *EFPs were obtained following stimulation of Tib (13), SP (6), PB (4), St (2), Su (3) and DP (3) nerves. All the nerves were stimulated at strengths 2-4xT*. The effects of the monoamines 5HT, DA and NA, both on DRPs evoked by intraspinal microstimulation and on the excitability of myelinated afferent fibers inferred by Wall's technique, were tested in 7, 7 and 6 experiments, respectively. EPSCs were recorded in 7 experiments and they were evoked by the stimulation of the Tib (7) and the Su (1) nerves at strengths 1.5-2 xT. 14 unidentified dorsal horn interneurons exhibiting EPSCs from the Tib (13) and Su (1) were recorded. The effects of 5-HT 10 µM were tested in all 14 interneurons, and the effects of mephenesin in 5 interneurons.

**Table 1:** Samples sizes of evoked responses, nerves stimulated, monoamines tested and stimulation strength.

		Nerves stimulated		Monoamines tested (10 µM)			Stimulation strength (xT)	
Experiments	Evoked responses	Tib	+ other	5HT	DA	NA	1.5–2	2–4
28	DRPs	20	23	28	24	23	-	26
19	EFPs	13	18	19	16	16	-	17
7	CAP	-	-	7	7	6	-	-
7	EPSCs	7	1	7	-	-	7	-

### Data Analysis

Since the onset of evoked-DRPs overlapped with afferent volleys and antidromically propagating dorsal root reflexes, it was not possible to reliably measure time to DRP onset for estimates of synaptic latency. DRP and short-latency EFP amplitude were measured from approximate onset to peak and expressed as percentage of control. Long-latency EFP amplitude was measured as the area between 100–300 ms. EPSC amplitude was measured as the area between the onset and 400 ms. The actions of bath-applied monoamines were measured after effects reached a steady-state (8–12 min after application). All data are expressed as percentage of the control value (mean ± SD), unless otherwise stated. For statistical comparison, we used Wilcoxon non-parametric test. All differences were considered significant if P<0.05.

## Results

### Effects of the monoamines on afferent-evoked responses following tibial nerve stimulation

#### DRPs

**Figure 3:**
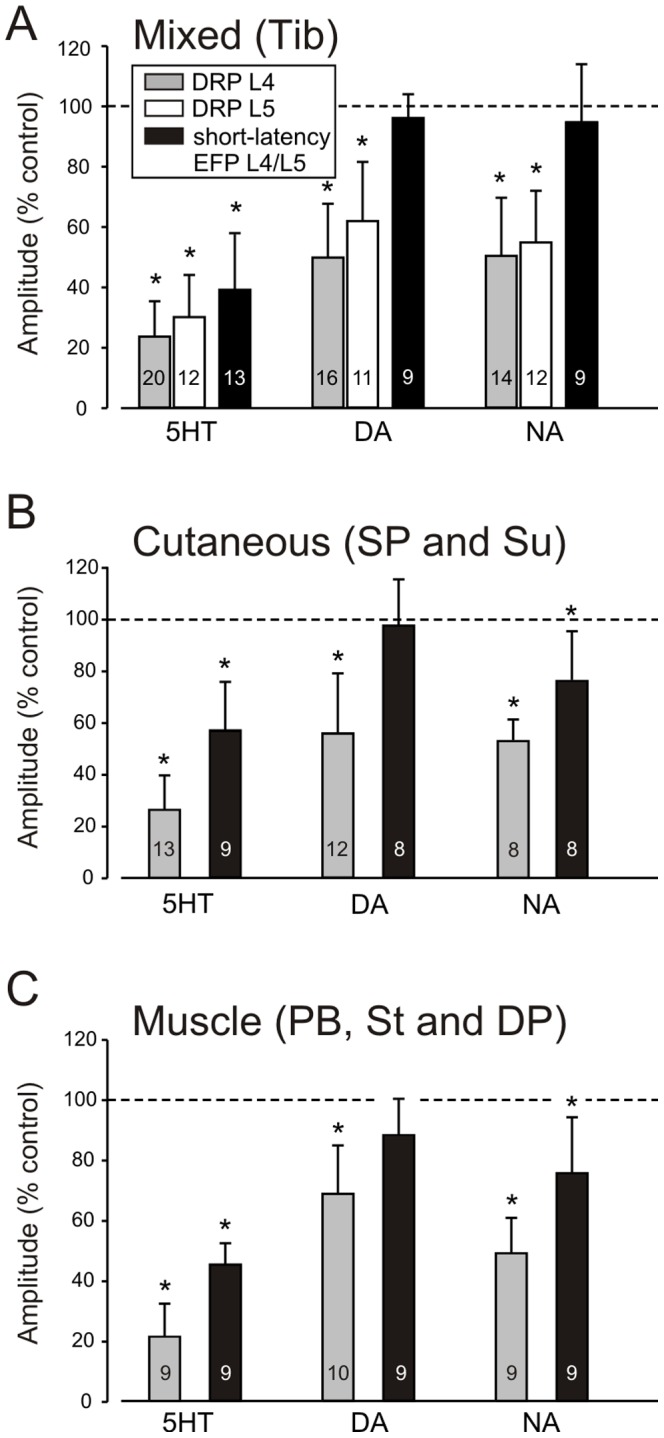
Summary graphs of the effects of monoamines on EFPs and DRPs evoked by stimulation of myelinated afferents. In A–C the bars represent the percentage inhibition (mean ± SD; samples sizes reported inside individual bars) of L4- and L5-DRP and the fast short-latency EFP amplitude with respect to control. **A**, L4-DRPs and L5-DRPs and EFPs evoked by stimulation of the Tib nerve. **B**, L4-DRPs and EFPs evoked by the stimulation of the cutaneous nerves SP and Su and **C**, L4-DRPs and EFPs evoked by the stimulation of the muscle nerves PBSt, St and DP. All the nerves were stimulated with strengths 2-4 xT. For the Tib nerve (A) the effects of 5HT, DA and NA on L4- and L5-DRPs are greatly reduced, as is the effect of 5HT on short-latency EFP (P<0.05). The effects of 5HT, DA and NA on cutaneous and muscle afferent stimulation-evoked DRPs is similarly reduced (B and C; P<0.05) while 5HT and NA also depress evoked EFPs (P<0.05).

Stimulation of the mixed cutaneous/muscle nerve Tib at 4xT evoked DRPs that reached peak amplitude at 56±16 ms from the earliest afferent volley and lasted 441±199 ms. All monoamines applied at 10 µM produced a pronounced reversible depression of Tib-evoked DRPs (Figure 2A–C). Comparable depressant actions were observed at L4 and L5 dorsal roots (Figure 3). For L4, 5HT depressed Tib-evoked DRPs to 23±11% of control values with DA and NA producing depressions to 50±18% and 50±19% of control values, respectively (Figure 3A). DRPs recovered to control values for 5HT and DA. There was only a partial recovery with NA even twenty-six minutes after wash out (Figure 2C).

#### Synaptic transmission

Only 5HT clearly depressed the short latency Tib-evoked EFPs (38±19% of control values; Figures 2 and 3A). As this short-latency response represents monosynaptic actions, 5HT reduced monosynaptic transmission. Longer latency EFPs were reversibly depressed by all monoamines (Figure 2Ai–Ci). When measured by area depression was to 20±11% (n = 21), 75±39% (n = 16) and 52±17% (n = 16) of control values, for 5HT, DA and NA, respectively.

**Figure 4:**
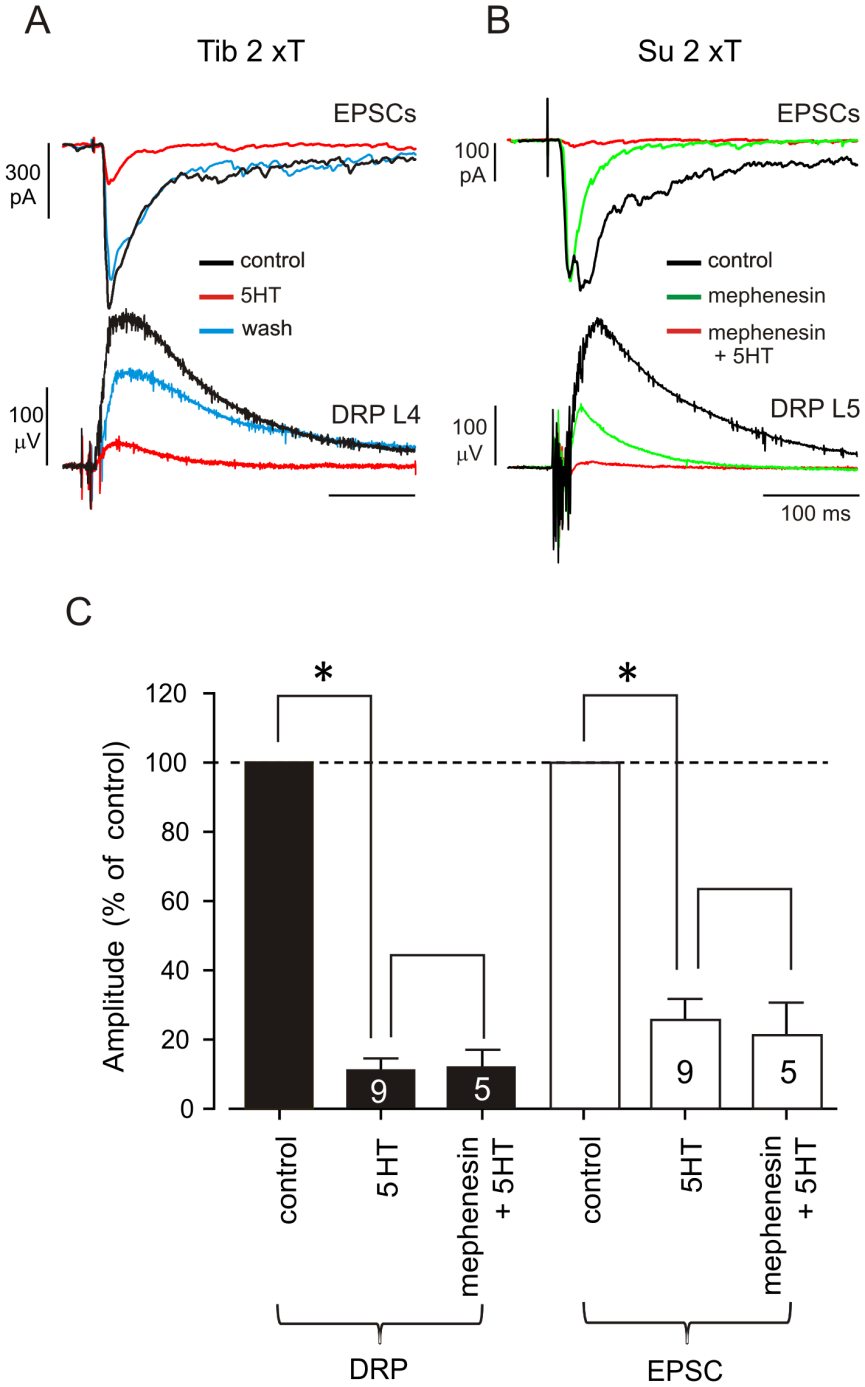
5HT depresses monosynaptic transmission of low threshold afferent fibers. **A**, EPSCs (*upper panel*) recorded on an unidentified L4 dorsal horn neuron and DRPs (*lower panel*) recorded at L4 dorsal root, before (black) during (red) and after (blue) bath application of 10 µM 5HT. Note the remarkable depression of EPSCs and DRPs, and the recovery after wash. **B**, EPSCs (*upper panel*) recorded on an unidentified L4 dorsal horn neuron and DRPs (*lower panel*) recorded at L5 dorsal root, before (black), during bath application of 1 mM mephenesin (green) and then 10 µM 5HT (red). Note that the monosynaptic component of the EPSC was virtually abolished after bath application of 5HT. **C**, summary graph of the depression (P<0.05) observed with 5HT and mephenesin + 5-HT on DRPs (*filled bars*) and EPSCs (*open bars*). The number of experiments is indicated inside graphed bars.

**Figure 5:**
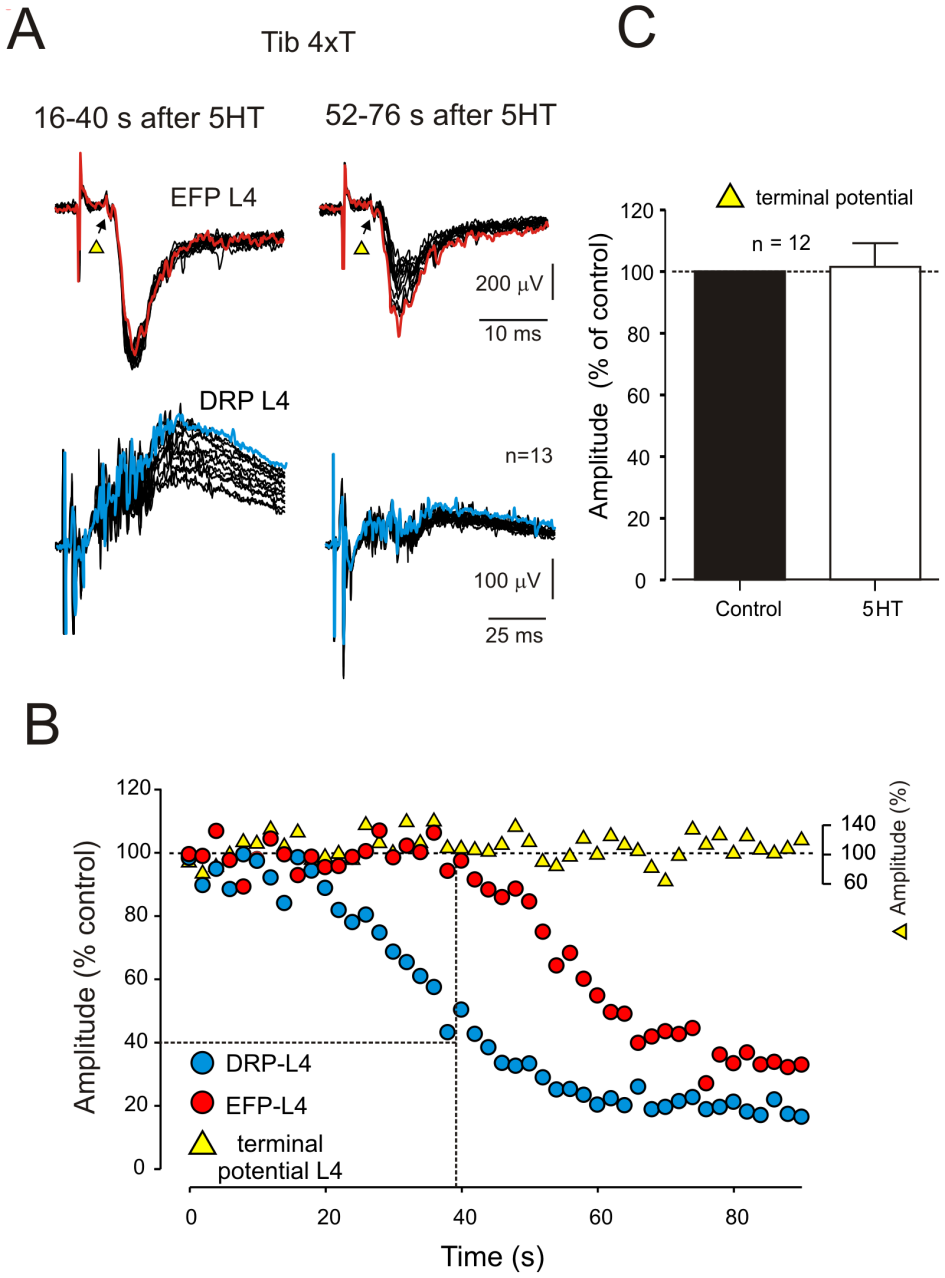
5HT depresses low threshold-evoked EFPs and DRPs with a different time course. **A**, EFPs (*upper traces*) and DRPs (*lower traces*) produced by the stimulation of the Tib nerve at 4xT and recorded at L4 dorsal horn and L4 dorsal root, respectively. Overlapped traces were recorded consecutively from 16–40 s (left panel) and from 52–76 s (right panel) after bath application of 5HT (10 µM). Red and blue traces correspond to the first recording for each period of time. Note that between 16–40 s after 5HT application DRPs are progressively depressed while the EFPs remain largely unaltered, and between 52–76 s the depression of DRPs reaches a plateau while EFPs are progressively depressed. Terminal potentials (arrows) are not affected by 5HT. **B**, comparison of the time course of decay of DRPs (*blue circles*) and EFPs (*red circles*). Note that depression of EFPs and DRPs is clearly phase-shifted. Vertical dashed line indicates the onset of EFP depression. Horizontal dashed line indicates DRP depression to 40% of control. Terminal potentials (TP, *yellow triangles*) remain unaltered in the presence of 5HT. **C**, summary graph of the effects of 5HT on the amplitude of terminal potentials respect to control. The effects were not statistically different (Wilcoxon test, P<0.05).

To further explore depression of monosynaptic transmission by 5HT, we recorded evoked EPSCs in deep dorsal horn interneurons. Like EFPs, EPSCs were also depressed by 5HT (to 25.6±18.8% of control, n = 9; Figure 4) including when the evoked synaptic responses were restricted to predominantly monosynaptic events with mephenesin (to 21.1±9.2% of control, n = 5; Figure 4). In comparison 5HT did not significantly alter membrane resistance (90.9±8.1%; n = 10). Together these observations support depression via presynaptic events on primary afferents. These presynaptic actions are likely mediated via activation of metabotropic receptors since action potential invasion into afferent terminals, measured as terminal potentials, was unaffected (101.5±26% of control, n = 12; Figure 5) [36].

The depression of the monosynaptic transmission (EPSCs and EFPs) by 5HT may be responsible for the statistically comparable percent reduction in DRP amplitude. If true, DRP reductions may have no bearing as a measure of changes in spinal circuits generating PAD, but rather simply reflect reduced sensory input to the spinal cord. However, most of the DRP depression occurred prior to changes in monosynaptic transmission. As shown in Figure 5, DRP amplitude depression began earlier and peaked earlier than EFP amplitude depression (see also Figure 2Aii). This was observed in 5/7 experiments. The plot in Figure 5B shows the DRP depressed to ∼40% of control amplitude prior to the start of EFP depression. Similar effects were also observed when simultaneously recording DRPs and EPSCs (n = 5).

### Depression of DRPs by 5HT, DA and NA was dose-dependent with similar efficacies

**Figure 6:**
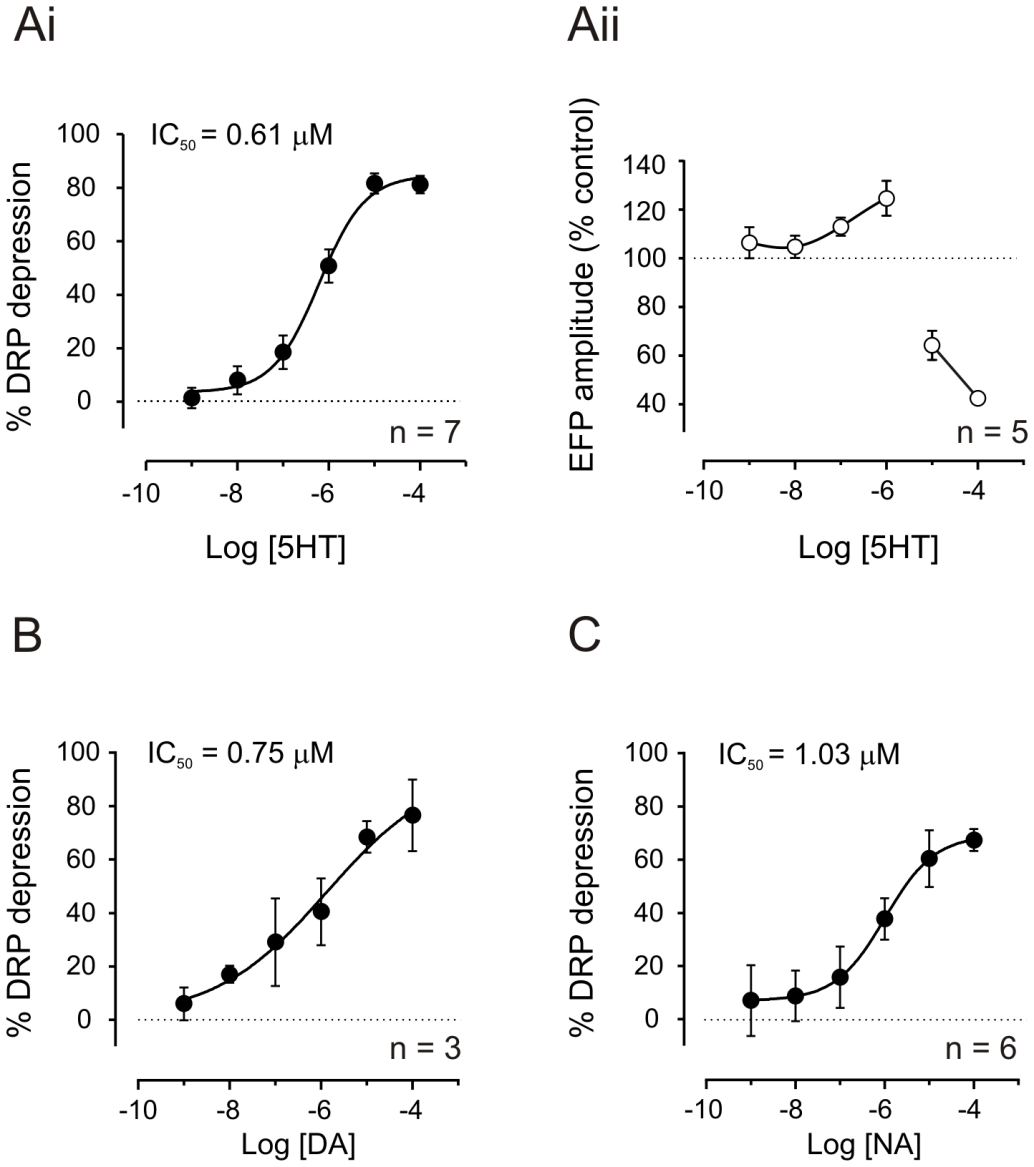
Monoamines depress DRPs in a dose-dependent manner while 5HT produces a biphasic effect on EFPs. Concentration-response curves of 5HT, DA and NA at cumulative concentrations of 0.001, 0.01. 0.1, 1, 10 and 100 µM, on DRPs (**Ai**, **B** and **C**) and EFPs (**Aii**) evoked by the stimulation of the Tib nerve at 4xT. DRPs are depressed in a dose-dependent manner by the three monoamines. The monosynaptic component of the EFPs is facilitated by 5HT at doses below 1 µM and inhibited with doses of 10 and 100 µM. Each point represents mean ± SE of amplitude respect to control. The curves are constructed from 7, 5, 3 and 6 experiments, as indicated. For the effects of 5HT, DA and NA on DRPs the pIC_50_s are 6.2±0.1, 6.1±0.7 and 6.0±0.1 (mean ± SE), and the corresponding IC_50_s are 0.6, 0.8 and 1.0 µM, as indicated. For the effect of 5HT on EFPs the curve was biphasic and IC_50_ values were not calculated.

The dose dependence of monoamine-induced DRP depression was examined. Cumulative concentration-response curves are shown in Figure 6. 5HT, DA and NA depressed evoked DRPs with similar potencies having IC_50_ values of 0.61 (n = 7), 0.75 (n = 3) and 1.03 (n = 6) µM, respectively. Interestingly, 5HT actions on EFPs were instead biphasic with a dose-dependent facilitation below 1 µM and depression at doses of 10 and 100 µM (Figure 6Aii). This is consistent with actions on different 5HT receptors with different affinities, and further differentiates modulatory actions seen at stimulated afferent synapses versus downstream spinal pathways that generate the DRP.

### Modulatory actions were comparable for responses evoked by stimulation of muscle and cutaneous nerves

**Figure 7:**
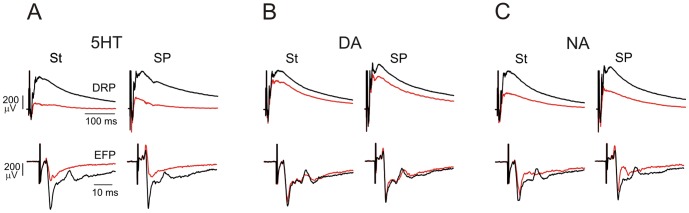
Comparing effects of the monoamines on muscle and cutaneous afferent-evoked DRPs and EFPs. **A–C**, DRPs (upper traces) and EFPs (lower traces) evoked by the stimulation of the muscle nerve St (left panels) and the cutaneous nerve SP (right panels), both with strengths 4xT. In A–C black traces show control recordings before, and red traces after 5 min of bath application of 5HT, DA and NA 10 µM, respectively. Note that 5HT, DA and NA depressed DRPs, but only 5HT and NA depressed the fastest components of low threshold-evoked EFPs.

We independently stimulated muscle and cutaneous nerves to compare modulation of their evoked DRPs and EFPs and observed comparable actions. Figure 7 shows examples following stimulation of the muscle nerve semitendinosus (St) and the cutaneous nerve superficial peroneal (SP). Overall, 5HT depressed both DRP and EFP amplitudes in all nerves tested (Figure 3). While DA and NA depressed DRPs evoked by all nerves, DA had no effect on EFPs, whereas NA had small but significant depressant actions on cutaneous- and muscle-evoked EFPs (Figure 3).

### 5HT, DA and NA have no effect on the excitability of low threshold afferent fibers

**Figure 8:**
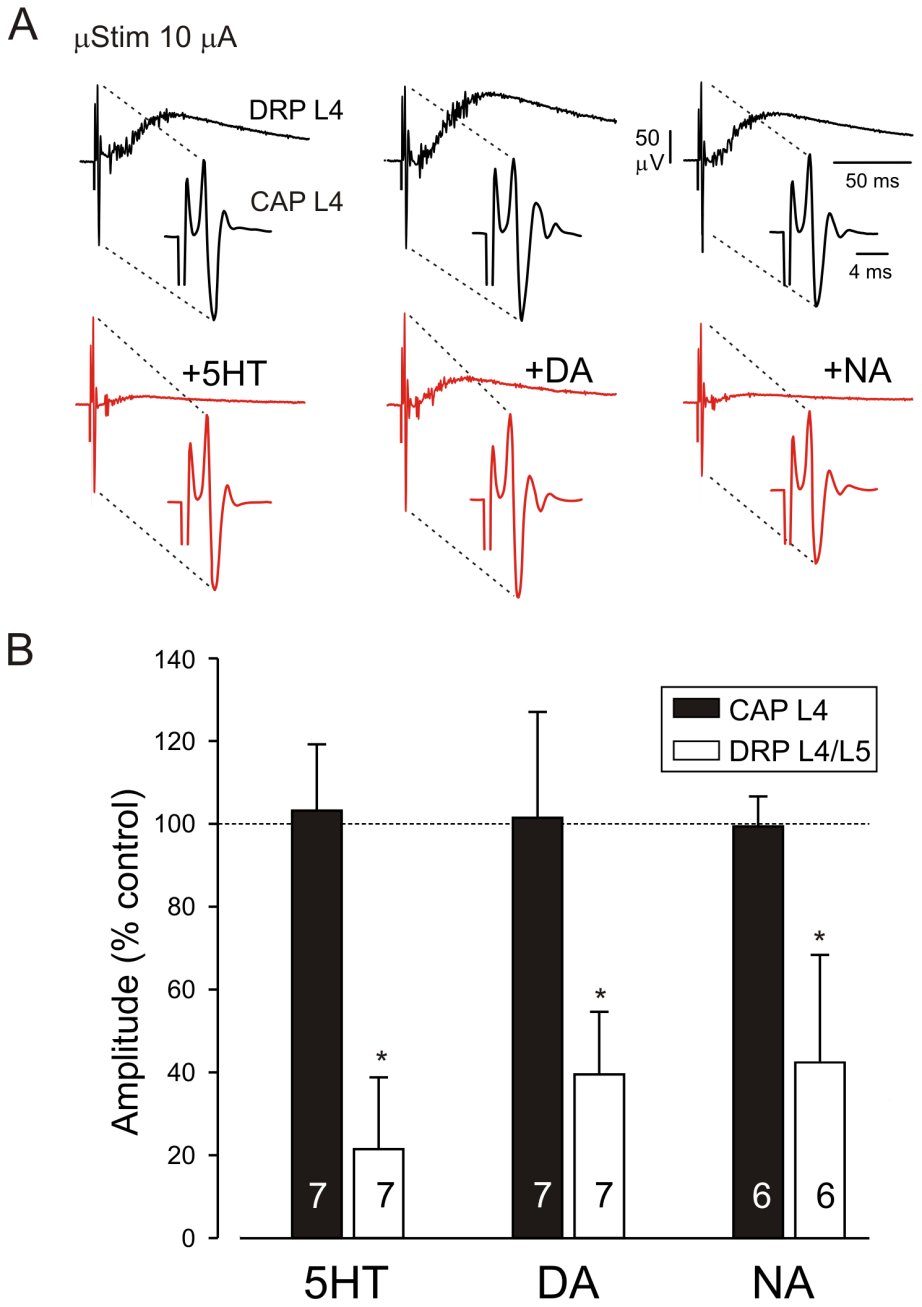
The monoamines have no effect on the excitability of myelinated afferent fibers. **A**, DRPs recorded at L4 dorsal root and evoked by intraspinal microstimulation (μStim) in control (upper traces) or after application of 10 µM 5HT, DA, or NA each (lower traces). Insets show expanded segments of upper traces. Note that DRPs are preceded by a short-latency compound action potential (CAP). The CAP evoked at the stimulus intensities used was always submaximal. Note that monoamines depressed intraspinal microstimulation evoked-DRPs but not the short-latency CAPs. Traces are averages of 16 consecutive events and the effects of 5HT, DA and NA. These effects were largely reversible during washout of each drug. **B**, summary graph of the effects seen. 5HT, DA and NA strongly depressed microstimulation-evoked DRPs (P<0.05) but not CAPs. The number of experiments is indicated inside the bars.

We used intraspinal microstimulation to test whether the monoamines directly modulated the excitability of afferent fibers. In the example shown in Figure 8A, intraspinal microstimulation evoked a short-latency antidromic compound action potential (CAP) in the L4 dorsal root that was followed by a DRP. Short-latency CAPs (expanded traces in insets) are produced by direct intraspinal activation of myelinated afferent fibers while DRPs reflect PAD via interneuronally-mediated synaptic actions on afferent terminals. Figure 8B shows that the monoamines did not have actions on the short-latency CAPs yet 5HT, DA, and NA reversibly depressed evoked DRPs to 21±17% (n = 7), 40±15% (n = 7) and 42±26% (n = 6) of control values, respectively (p<0.05). This magnitude of DRP depression was comparable to that seen on DRPs evoked by peripheral nerve stimulation (cp. Figure 3).

These experiments demonstrate that monoamine modulatory actions on the DRP are independent of electrical excitability changes in the primary afferents terminals which could occur, for example, via activation of ionotropic 5-HT_3_ receptors. When combined with earlier evidence that DRP depression precedes depression in monosynaptic transmission, spinal interneurons are implicated as the predominant site for monoaminergic regulation of DRP amplitude.

## Discussion

The present results demonstrated that 5HT, DA and NA have common depressant actions on DRPs evoked by stimulation of myelinated cutaneous and muscle afferents. The magnitude of these observed actions was dose-dependent. Evidence supporting depression of afferent transmission was also seen for 5HT and NA. Recently, 5HT, DA and NA were reported to depress DRPs and afferent transmission and DRPs produced by visceral afferent stimulation [5]. Together, it is clear that monoaminergic descending systems have broad modulatory control over somatosensory input.

Afferent activity provides a continuous stream of movement-dependent feedback to reinforce and refine ongoing behaviours, and can also reconfigure, initiate, and terminate motor tasks [37]. The monoamine transmitters are clearly involved in modulating the performance of spinal motor systems, yet little is known of their actions on the cutaneous and muscle proprioceptive afferents recruited during these behaviours. Here we found that 5HT, DA and NA potently (IC_50_ values ∼0.6 to 1.0 µM) and efficaciously reduced DRP amplitudes evoked by stimulation of low-threshold myelinated afferent fibers (76, 44 and 50% reductions at 10 µM, respectively). The DRP depression produced by 5HT and NA supports and extends previously observed actions following dorsal root stimulation of unknown primary afferents [11] and further broadens the analysis to include the actions of DA. Inasmuch as the DRP is a reflection of PAD-mediated presynaptic inhibition, the monoamine transmitters greatly reduce cutaneous and proprioceptive afferent-evoked presynaptic inhibition. Since a dominant form of presynaptic inhibition is via autogenic negative feedback [6], that is, back onto the same stimulated myelinated afferents, the monoamines may limit depression of sensory transmission in recruited afferents that fire repetitively during ongoing movements. Using the experimental procedure employed, we could not determine whether the DRP produced by stimulation of myelinated afferents included actions on non-myelinated afferents, although it is known that stimulation of cutaneous Aβ afferents can produce PAD on C afferent fibers [6].

DRPs evoked by stimulation of selective muscle and cutaneous nerves were similarly depressed by monoamines (applied at 10 µM). As DRP depression reflects reduced afferent presynaptic inhibition, this suggests that monoamines can broadly facilitate the actions of proprioceptive feedback during movement, consistent with the view that monoamines facilitate circuits engaged in motor behaviors [3]. 5HT in particular also depressed monosynaptic EFPs and EPSCs. This supports earlier observations of reduced afferent monosynaptic transmission in deep dorsal horn neurons [2], but does not exclude additional postsynaptic actions. Thus, while interneurons involved in the circuitry responsible for PAD are generally inhibited by the monoamines, proprioceptive input strength can also be concomitantly reduced. The net effect of modulation at both sites could be to permit ongoing recruited afferents to exert weaker actions that are nonetheless less susceptible to a history-dependent depression via PAD-mediated presynaptic inhibition.

It is unlikely that the observed reduction of DRPs is simply a consequence of reduced monosynaptic transmission of stimulated afferent fibers as EFP amplitude reductions developed more slowly. Thus, the DRP depression also includes modulatory actions further downstream, either on interneurons mediating PAD, and/or on the last-order synapse producing PAD. 5HT may also facilitate monosynaptic transmission at lower doses (≤1.0 µM) suggesting that 5HT bi-directionally modulates transmission in a concentration dependent manner. This is presumably mediated by 5HT receptor subtypes with different agonist affinity. As lower 5HT doses facilitate monosynaptic strength while still depressing DRPs, the net consequence would be an amplification of central synaptic actions with less history-dependent depression via presynaptic inhibition. Overall, the monoamines, and in particular 5HT, can modify afferent processing by at least three independent mechanisms, two increasing (↓DRP; ↑EFP) and one decreasing (↓EFP) afferent transmission.

As EFP and DRP reductions are temporally and spatially dissociable, physiological mechanisms could exist for their independent control by descending systems. For example, the strong 5HT depression of the short-latency muscle afferent-evoked EFP likely reflects reduced population Ia synaptic transmission in the dorsal horn, consistent with 5HT depressant actions on Ia input to motoneurons [22]. Yet, local iontophoretic application of 5HT can instead facilitate group I muscle afferent input to dorsal horn interneurons [1]. As we also observed EFP facilitation at lower doses of 5HT, the control of activity of interneurons producing PAD may depend on the strength of descending drive and the presence of 5HT receptors with varying affinity [3].

### On the depression of the second short-latency and the long-latency components of EFPs

Although in this study we cannot discriminate whether the second short-latency component of the EFP is produced by group II or other slower conducting myelinated fibers or to polysynaptic pathways, the depression of this component by all monoamines when stimulating muscle nerves is consistent with observations on group II field potentials in the cat by NA [1], [24], [25], and DA agonists [26]. As the onset of this EFP begins after DRPs are already recruited, it cannot be associated with activation or modulation of the early part of the DRP.

The long-latency slow decaying EFP is also suppressed by all monoamines. Since stimulus intensity for afferent recruitment was predominantly below recruitment threshold for C/group IV afferents, these events are initiated by lower threshold afferents. The slow decaying EFP may be due to recruitment of polysynaptic pathways, activation of the kinetically slower NMDA receptor [38] and likely also reflects the PAD field recorded intraspinally since it matches the time course of DRPs [39].

### Putative mechanisms

**Figure 9:**
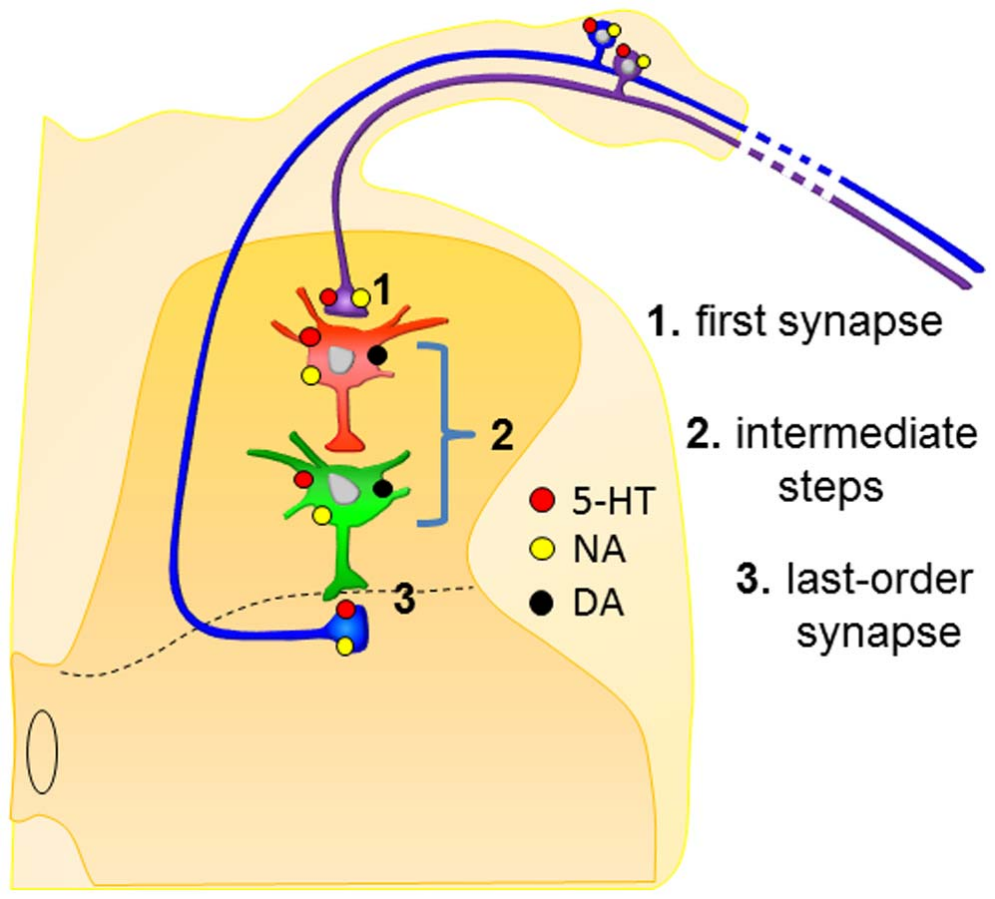
Summary interpretation of changes observed. Drawing highlights putative sites of action of the monoamine transmitters. **1**. The short-latency EFP reflects monosynaptic transmission from primary afferents, which is reduced by 5HT and NA, but not DA. As this occurs in the absence of changes in excitability of primary afferents (no change in CAP amplitude), actions are likely exerted via activation of 5HT and NA metabotropic receptors (red and yellow dots, respectively). These receptors will act on signal transduction pathways to reduce synaptic transmission and/or reduce glutamate receptor responsiveness on postsynaptic interneurons. **2**. The intraspinal circuit responsible for PAD may involve one or more interposed interneurons, as depicted in the schematic. Monoamines may reduce the excitability of these interneurons and/or reduce transmission between first and higher-order interneurons by activation of 5HT, NA and DA receptors (red, black and yellow dots, respectively) so that fewer interneurons are recruited to produce PAD. **3**. 5HT, DA, and NA may act at the last order GABAergic axo-axonic synapse leading to reduced activation of the GABA_A_-like receptors responsible for producing PAD.

Bulbospinal monoamines activate numerous metabotropic receptor subtypes that couple to signal transduction pathways. Since ligand- and voltage-gated channels are modulated through signal transduction pathways, it is likely that the monoamines modify the function of many membrane channels that control excitability of neurons. Most or all spinal neurons and primary afferents contain at least some monoamine receptor subtypes, and most receptors are expressed in subpopulations of primary afferents and dorsal horn neurons [4]. Hence, monoaminergic modulatory actions may occur on all neuronal elements in the pathway involved in generating PAD; directly on afferent fibers giving PAD, interneurons mediating PAD, and on fibers receiving PAD. A summary figure of possible sites of action is shown in Figure 9, and discussed with examples provided below. The contribution of identified monoamine receptors on specific sensory fiber terminals or characterized interneurons mediating PAD remains to be determined.

### A. Presynaptic mechanisms

As the terminal potential amplitude evoked by stimulation of lower-threshold afferents was unchanged, it is unlikely that the invasion of action potentials in presynaptic axonal terminals was altered [36]. The lack of effect supports changes independent of terminal excitability, both in the stimulated afferents, and in those targeted to generate the DRP. Thus, the reduction by the monoamines of the short-latency monosynaptic EFPs occurs as a result of reduced synaptic efficacy of the afferents, and/or a reduced response of postsynaptic receptors (e.g. glutamate receptors).

The monoamines also did not alter the amplitude of intraspinal stimulation-evoked antidromic CAPs (Wall's technique). The lack of a change in CAPs excludes modulation of synaptic efficacy via mechanisms related to changes in polarization of the afferent terminals, as for example would be expected with direct activation of ionotropic 5HT_3_ receptors found on axon terminals of higher-threshold afferents [8], [40]. Thus, observed actions are most likely via 5HT metabotropic receptor-mediated actions on the afferent fibers, or interposed interneurons. This is supported by the fact that iontophoretic application of NA and 5HT produces no changes in the intraspinal threshold of identified group Ia afferents [18]. As locally applied NA and its agonists have no effect on low-threshold cutaneous afferents evoked responses on spinal neurons [14], [15], [16], the DRP depression produced by NA is likely to be via metabotropic presynaptic actions.

### B. Postsynaptic mechanisms

Since primary afferents are glutamatergic, depression of postsynaptic glutamate receptor activity would reduce EFP amplitude. As D_2-like_, 5HT_1_ and α_2_-adrenergic are G_i_-coupled receptors that reduce phosphorylation of non-NMDA receptors, activation of these postsynaptic monoamine receptors could depress EFP amplitude via glutamate receptor depression [41].

The monoamines may also reduce the membrane excitability of interposed interneurons. For example, NA depresses postsynaptic lamina II neurons via *α*_2_-adrenoceptors acting on an outward K^+^ current [42], [43]. Similarly, DA activates K^+^ channels via D_2_-like receptors on substantia gelatinosa neurons [44]. Regarding 5HT, stimulation of the raphe magnus inhibits activity in dorsal horn neurons presumably via spinal 5HT_1_ receptors [45] and depression of group II afferent activity includes 5HT_3_ receptor actions likely in excitatory interneurons [27].

### C. Actions on primary afferents producing PAD

As PAD is thought to be predominantly generated by activation of GABA_A_ receptors, the monoamines could depress PAD via modulation of GABA_A_ receptor activity on afferent fibers. The GABA_A_ receptor has multiple phosphorylation sites to support monoaminergic modulatory actions [46]. For example, 5HT_1B_ receptors can depress GABA_A_ receptor-mediated currents [47] and may reduce GABA release [48]. Both actions would support a reduction in DRP amplitude. D_1-like_ receptors have also been reported to depress activation of GABA_A_ receptor currents [49]. Regarding NA, some α_2_-adrenergic receptor immunoreactivity is found in the axon terminals of GABAergic neurons [50]. As GABAergic interneurons mediating PAD may express these receptors, NA may reduce the synaptic efficacy of inhibitory interneurons mediating PAD. Rather than modulate GABA_A_ receptors, actions on signal transduction pathways controlling the NKCC1 Cl^-^ cotransporter could reduce PAD by reducing the Cl^-^ driving force. Indeed, 5HT_2A_ receptor activation was recently shown to increase synaptic inhibition in motoneurons via a KCC2 Cl^-^ cotransporter dependent hyperpolarization of the Cl^-^ reversal potential [51].

## Conclusions

In conclusion, we used the isolated *in vitro* mouse spinal cord preparation to demonstrate that the monoamines have widespread and common depressant actions on PAD evoked following stimulation of myelinated afferents from either cutaneous of muscle nerves. 5HT and NA also depress monosynaptic afferent transmission in the fastest conducting myelinated afferents. The depressant actions of 5HT on PAD and synaptic transmission are temporally discernible, and possibly mediated via activation of different receptor subtypes. An *in vitro* approach with selective afferent recruitment, intact circuitry, enhanced pharmacological precision, and stable whole-cell recordings from synaptically- and/or molecularly identified interneurons promises to yield comprehensive insight into modulatory mechanisms controlling somatosensory information processing.
